# The latest research progress on the clinical application of nemonoxacin

**DOI:** 10.3389/fmed.2025.1671640

**Published:** 2025-09-10

**Authors:** Hui Yin, Yingchun Shao, Fusheng Sun

**Affiliations:** ^1^Qingdao Hospital, University of Health and Rehabilitation Sciences (Qingdao Municipal Hospital), Qingdao, China; ^2^Pharmacy Department of Rizhao Central Hospital, Rizhao, China

**Keywords:** nemonoxacin, antibacterial activity, immune regulation, infectious diseases, drug resistance

## Abstract

Nemonoxacin is a novel non-fluorinated quinolone antibacterial agent, characterized by a broad antibacterial spectrum, strong antibacterial activity, low protein binding rate, high oral bioavailability, and extensive tissue distribution. Its unique pharmacological properties confer high clinical application value. In the context of bacterial resistance, this article aims to provide a comprehensive review of nemonoxacin’s antibacterial activity, immunomodulatory effects, and its application in infectious diseases as well as in patients with hepatic or renal insufficiency, with the goal of offering more drug options for clinical treatment and providing a reference for future clinical research and widespread application.

## 1 Introduction

Nemonoxacin, as the world’s first fluorine-free quinolone antibacterial drug (1), was developed by Procter & Gamble in the United States. Its clinical development was subsequently advanced through the collaboration between Zhejiang Medicine Co., Ltd. and TaiGen Biotechnology Co., Ltd. in Taiwan, marking an innovative breakthrough in the new generation of quinolone drugs. Its oral formulation, the nemonoxacin malate capsule, was first launched in Taiwan, China in March 2014 (2), and subsequently received the approval number and new drug certificate from the China food and drug administration (CFDA) in May 2016. Its injectable formulation (nemonoxacin malate sodium chloride injection) also passed the review by the national medical products administration (NMPA) in June 2021, completing the clinical layout of the formulation and was approved for market launch (3).

Pharmacokinetic studies have demonstrated that nemonoxacin is rapidly and completely absorbed after oral administration, with peak plasma concentrations achieved within 1–2 h post-dose (4). The absolute bioavailability is nearly 100%, and the elimination half-life exceeds 10 h, supporting a once-daily dosing regimen (5). The drug is widely distributed in target organs such as lung tissue, bronchial mucosa, bone, and the urinary system, with concentrations in the alveolar epithelial lining fluid reaching over four times those in plasma (6, 7). Additionally, the plasma protein binding rate is only about 16%, suggesting potential advantages in penetrating infection sites and exerting antibacterial activity. Furthermore, the drug is primarily excreted through the kidneys, with approximately 60%–70% of the administered dose excreted unchanged in the urine within 72 h (8), providing a pharmacokinetic basis for dose adjustment in patients with renal impairment.

With the expansion of clinical applications and in-depth basic research, the multiple mechanisms of action of nemonoxacin have gradually been elucidated, including its activity against multidrug-resistant bacteria (9), immunomodulatory effects (10), and applicability in complex infectious diseases (11). Therefore, this article systematically reviews its antibacterial activity, impact on bacterial resistance and clinical advantages, immunoregulatory effects, clinical application value in infectious diseases, and explores personalized medication strategies based on the pharmacokinetic characteristics of patients with hepatic or renal insufficiency. The aim is to provide scientific references for rational clinical drug use and subsequent research directions, to address the challenges of antimicrobial resistance and optimize treatment outcomes.

## 2 The impact of nemonoxacin on bacterial antibacterial activity

### 2.1 Chemical structure and antimicrobial spectrum of nemonoxacin

**Figure 1:**
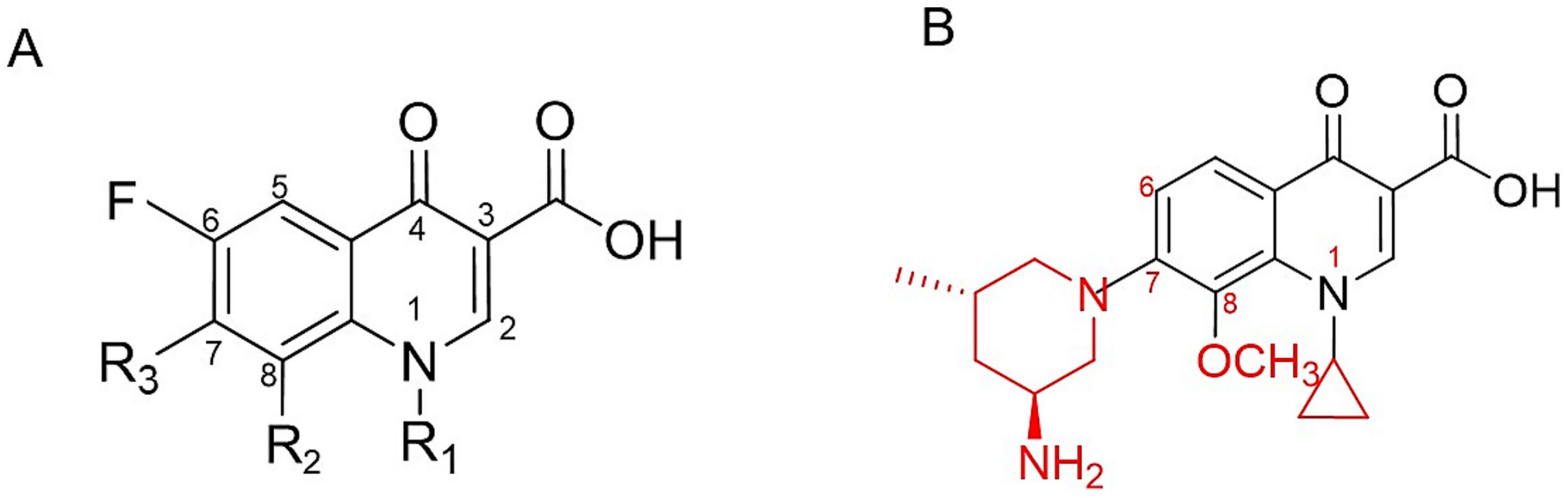
Chemical structure modification of nemonoxacin based on the fluoroquinolone core. **(A)** Structure of the fluoroquinolone core; **(B)** chemical structure of nemonoxacin.

The chemical formula of nemonoxacin is C_20_H_25_N_3_O_4_. Compared with traditional fluoroquinolones, the fluorine atom at the C-6 position is removed from the fluoroquinolone core structure, and an aminomethylpiperidine ring is introduced at the C-7 position, while retaining the cyclopropyl group at the N-1 position and the methoxy group at the C-8 position, which are consistent with those of noxifloxacin (Figure 1). The chemical structural modifications not only reduce adverse reactions but also expand the antibacterial spectrum, covering aerobic Gram-positive bacteria (G^+^ bacteria), Gram-negative bacteria (G^−^ bacteria), some anaerobic bacteria, and atypical pathogens (12).

### 2.2 Advantages of nemonoxacin

**Figure 2:**
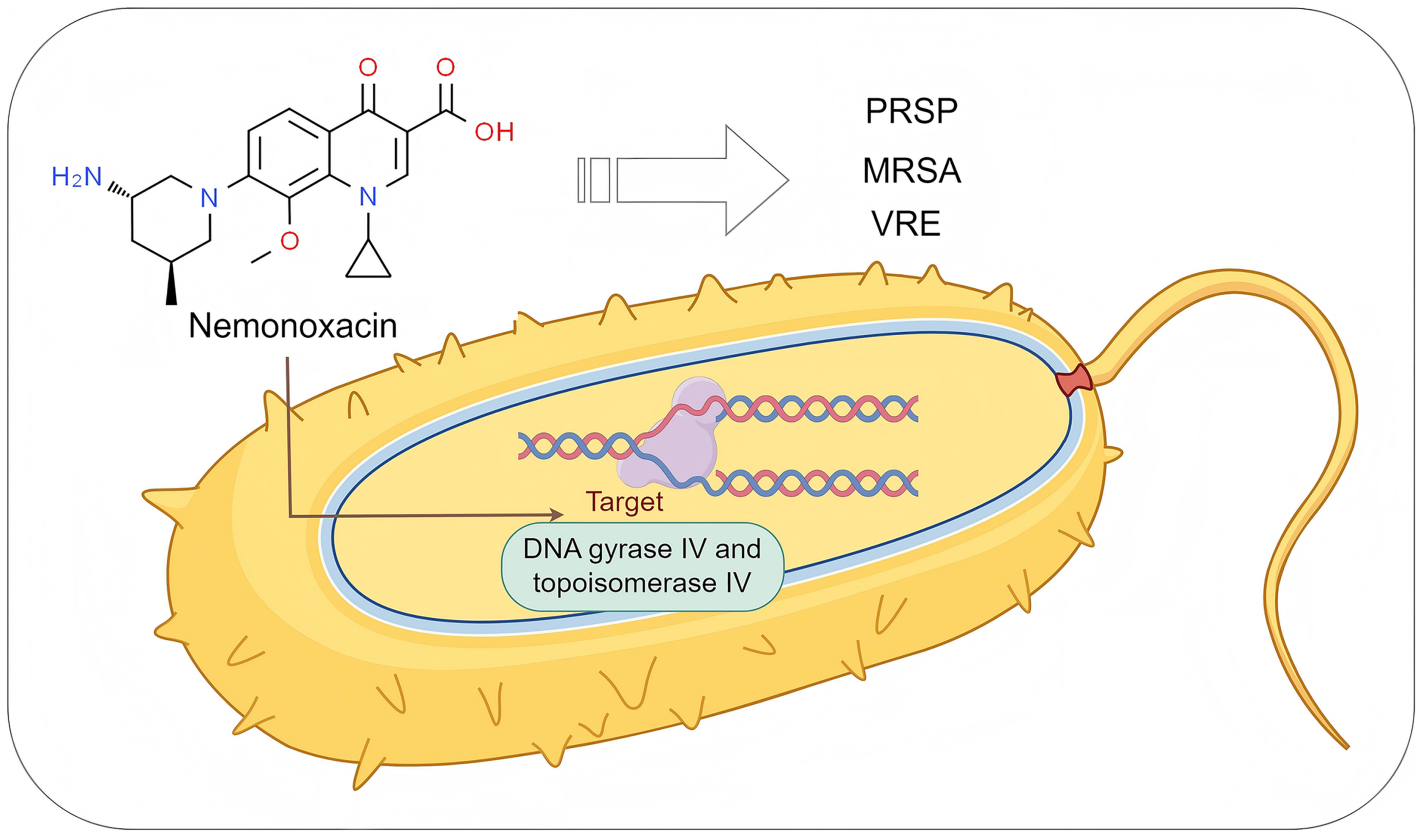
The antibacterial mechanism of nemonoxacin. PRSP, penicillin-resistant *S. pneumoniae*; MRSA, methicillin-resistant *S. aureus*; VRE, vancomycin-resistant enterococci. Created with Figdraw (www.figdraw.com).

Compared to traditional fluoroquinolone antibiotics, nemonoxacin demonstrates significant clinical advantages through structural optimization (non-fluorine substitution at the C-6 position and introduction of a methoxy group at the C-8 position) and innovation in its mechanism of action (Table 1). The introduction of a methoxy group at the C-8 position significantly enhances the dual inhibitory effects on bacterial DNA gyrase IV and topoisomerase IV (Figure 2), thereby resulting in a broader antibacterial spectrum and reduced selective pressure for resistance mutations (13). It exhibits high sensitivity particularly against G^+^ bacteria, including penicillin-resistant *Streptococcus pneumoniae* (PRSP) (14), methicillin-resistant *Staphylococcus aureus* (MRSA) (15), and vancomycin-resistant *enterococci* (VRE) (16). For example, its MIC50/90 (0.06/2 mg/L) against MRSA is lower than that of traditional fluoroquinolones such as levofloxacin, and its MIC50/90 (4/16 mg/L) against VRE is also significantly superior to levofloxacin (4). Meanwhile, it shows prominent advantages against atypical pathogens. For instance, its antibacterial activity against both macrolide-sensitive and macrolide-resistant *Mycoplasma pneumoniae* is stronger than that of moxifloxacin and levofloxacin (17); its activity against *Clostridium difficile* and *Mycobacterium abscessus* is superior to that of clinically commonly used fluoroquinolones such as levofloxacin and ciprofloxacin (18–20); its *in vitro* activity against *Helicobacter pylori* is 1 to 2 times stronger than that of ciprofloxacin and the like (21); and its MIC against *Nocardia* species is much lower than that of ciprofloxacin, levofloxacin, moxifloxacin, and gemifloxacin (22, 23).

**Table 1:** The clinical advantages of nemonoxacin.

Characteristic	Nemonoxacin	Traditional fluoroquinolones (levofloxacin/moxifloxacin/ciprofloxacin)	Advantage manifestation	References
Chemical structure	No fluorine atom at C-6 position C-8 methoxy group	C-6 position containing a fluorine atom	Reduce fluorine-related adverse reactions Enhance the antibacterial activity of G^+^ bacteria	(3, 5)
Antibacterial spectrum	Coverage of G^+^ bacteria (such as *S. pneumoniae*, *S. aureus*, *S. epidermidis*, *Enterococcus*, etc.) G^−^ bacteria (*H. influenzae*, *E. coli*, *K. pneumoniae*, *P. aeruginosa*) Atypical pathogens (such as *M. pneumoniae*, *Legionella pneumophila*), and anaerobic bacteria	The activity against most G^−^ bacteria is comparable to or superior to that of Nemonoxacin It is less effective against most G^+^ bacteria, atypical pathogens, and anaerobic bacteria compared to nemonoxacin Strong activity against *Mycobacterium tuberculosis*	Covering common pathogens of community-acquired infections while also addressing resistant G^+^ bacteria (such as PRSP, MRSA, MRCNS, VRE) and macrolide-resistant atypical pathogens (such as MRMP)	(13, 20, 25)
Drug resistance mechanism	Dual inhibition of DNA gyrase and topoisomerase IV	The key regions determining bacterial resistance to this class of drugs are primarily located on the gyrA, gyrB, parC, and parE genes, especially gyrA and parC	No parC gene mutation, more advantageous against parC mutant resistant bacteria Enhancing the antibacterial activity and resistance mutation prevention of vancomycin against MRSA	(13)
Adverse reaction	The incidences of phototoxicity and cardiotoxicity were significantly lower than those of fluoroquinolones	The risks of phototoxicity (such as moxifloxacin) and prolonged Q-T intervals (such as ciprofloxacin) are relatively high	The adverse reactions are few and slightly reversible Good tolerance High cardiac safety	(5, 64)

Compared with non-quinolone drugs, nemonoxacin also has obvious advantages: its MIC50 (0.06 mg/L) against MRSA is lower than that of tigecycline and tedizolid (4), and its MIC90 (1 mg/L) is lower than that of teicoplanin (2 mg/L) (24); its MIC50/90 (0.06/2 mg/L) against MRSA is lower than that of vancomycin, norvancomycin, linezolid, and ceftaroline (4); its MIC50/90 (4/16 mg/L) against VRE is also significantly superior to ampicillin, high-level gentamicin, and nitrofurantoin (4); its MIC50/90 (1/8 mg/L) against *C. difficile* is lower than that of clindamycin, with MIC50 equivalent to daptomycin and MIC90 lower than rifaximin (19); against *Nocardia* species, nemonoxacin shows better antibacterial activity compared with carbapenems, linezolid, vancomycin, tigecycline, trimethoprim-sulfamethoxazole, amikacin, and ceftriaxone (22, 23). Therefore, nemonoxacin has significant comprehensive advantages over traditional fluoroquinolones and non-quinolone drugs in terms of antibacterial activity against G^+^ bacteria, atypical pathogens, and special pathogens such as *C. difficile* and *M. abscessus*, providing a better option for the treatment of related infections.

### 2.3 The impact of nemonoxacin on bacterial resistance

#### 2.3.1 Antibacterial activity and coverage advantage against resistant bacteria

*In vitro* studies have shown that the MIC range of nemonoxacin (0.06–0.25 μg/mL) against *S. pneumoniae* is significantly lower than that of levofloxacin (0.5–1 μg/mL) and moxifloxacin (0.125–0.5 μg/mL), and it remains active against moxifloxacin-resistant MRSA strains (25). Chen et al. (25) further confirmed that nemonoxacin remains effective against moxifloxacin-resistant isolates among the major epidemic lineages of MRSA in Taiwan (ST8, ST59, ST239, and CC45). In addition, Yang et al. (13) discovered through *in vitro* resistance induction experiments that the MIC values of nemonoxacin against *S. aureus* and *Enterococcus* spp. did not significantly increase during prolonged exposure, while the MIC for *S. pneumoniae* only increased fourfold. Moreover, its resistance mutation frequency was significantly lower than that of ciprofloxacin, levofloxacin, and moxifloxacin, indicating that nemonoxacin has a high resistance barrier against G^+^ bacteria (12).

#### 2.3.2 Dual-target mechanism and synergistic anti-resistance strategy

Nemonoxacin significantly reduces the risk of resistance caused by single-site mutations through its dual-target mode of action, simultaneously inhibiting DNA gyrase (GyrA/B) and topoisomerase IV (ParE). Huang et al. (15) demonstrated that the combination of nemonoxacin and vancomycin (MIC = 2 μg/mL) synergistically enhances bactericidal activity against MRSA (FICI ≤ 0.5). By blocking both cell wall synthesis and DNA replication, this dual mechanism reduces the selective pressure for resistant mutants. This combined strategy not only improves antimicrobial efficacy but also delays the development of resistance through target complementarity, providing an optimized treatment regimen for multidrug-resistant bacterial infections such as MRSA.

## 3 The immunomodulatory effects of nemonoxacin

Nemonoxacin, as a novel non-fluorinated quinolone antibacterial agent, has demonstrated its immunomodulatory effects in both *in vivo* and *in vitro* experiments. Studies have shown that nemonoxacin exhibits significant immunomodulatory and protective effects against lipopolysaccharide (LPS)-induced inflammatory responses. Specifically, it effectively suppresses LPS-induced macrophage inflammatory responses by downregulating the expression levels of pro-inflammatory cytokines (such as IL-6 and TNF-α) while upregulating the expression of anti-inflammatory factors (such as IL-10), thereby preventing excessive immune activation in the host (10). Furthermore, nemonoxacin can enhance the bacterial phagocytic ability of macrophages, further regulating immune responses and contributing to the improvement of immune function status in patients with severe infectious diseases (26). Notably, the immunomodulatory effects of nemonoxacin are currently limited to animal studies, and no sufficient literature on clinical validation has been retrieved. Therefore, more rigorously designed, large-sample studies are urgently needed to further confirm its immunomodulatory efficacy in clinical patients.

## 4 The application value of nemonoxacin in infectious diseases

**Figure 3:**
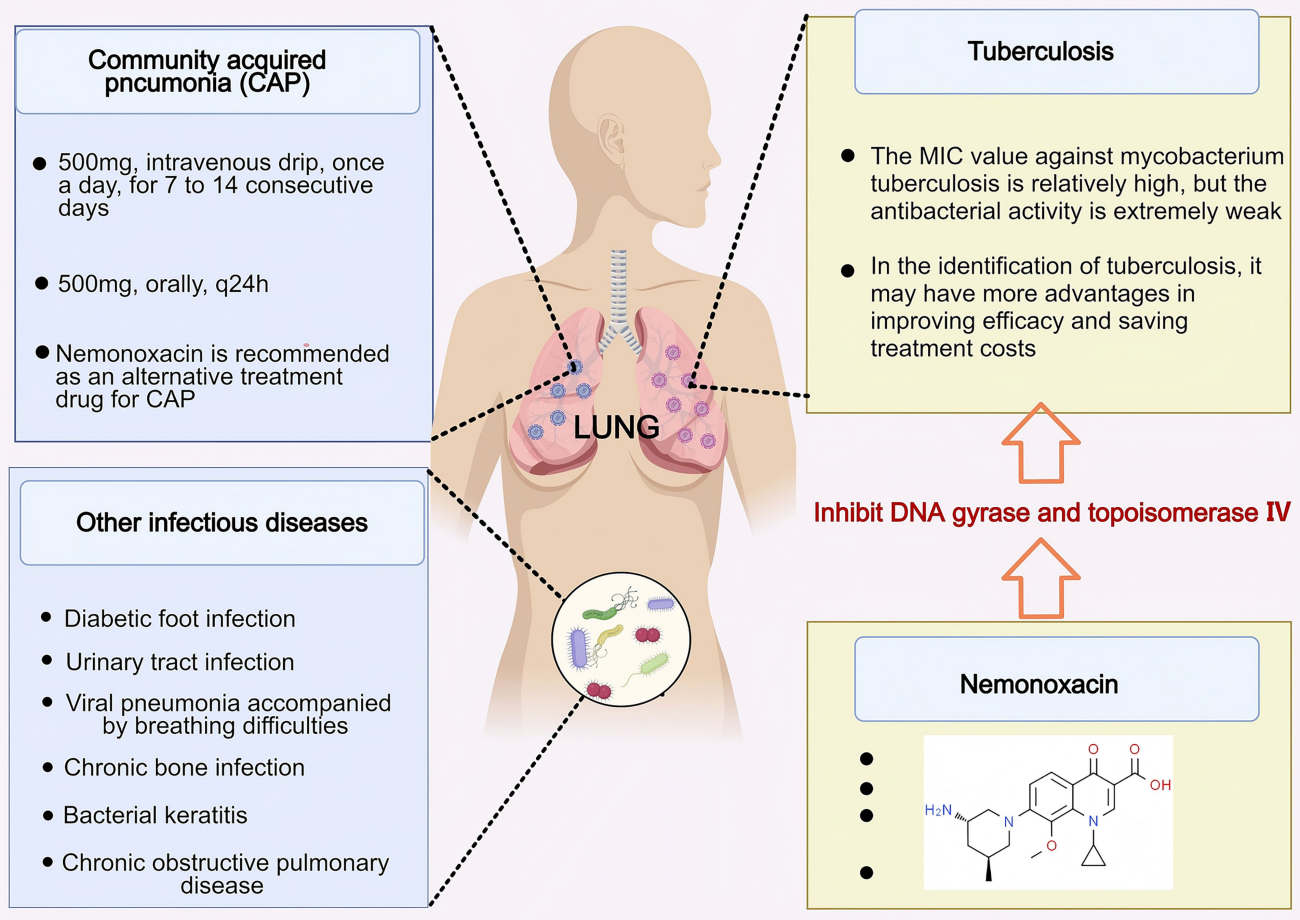
The application value of nemonoxacin in infectious diseases. Nemonoxacin has therapeutic effects on infectious diseases such as CAP, DFI, AECOPD, UTI, patients with viral pneumonia accompanied by dyspnea and hypoxia, and local treatment of bacterial keratitis. Created with MedPeer (medpeer.cn).

Nemonoxacin exhibits broad-spectrum antibacterial activity and is primarily used for the treatment of community-acquired pneumonia (CAP) in adults post-market. With the continuous advancement of clinical research, it has also demonstrated significant clinical application value in other infectious diseases (Figure 3).

### 4.1 Clinical applications in CAP

CAP is one of the most common infectious diseases, a leading cause of hospitalization and death, and imposes substantial healthcare costs (27). Numerous clinical trials and post-marketing surveillance studies have found that the safety, clinical efficacy, microbial treatment success rate, and tolerability of nemonoxacin in treating adult CAP patients are not inferior to those of fluoroquinolone antibiotics. A multicenter, randomized, double-blind, double-dummy, parallel-controlled phase III clinical trial has demonstrated that the clinical efficacy and safety of nemonoxacin (500 mg, ivgtt, qd, for 7–14 days) in the treatment of hospitalized adult patients with CAP are comparable to those of levofloxacin (500 mg, ivgtt, qd, for 7–14 days) (28). The adverse reactions (such as local reactions at the infusion site, nausea, Q-T interval prolongation, etc.) were mostly mild and reversible (28). Systematic reviews and meta-analyses further support that the clinical cure rate, incidence of adverse reactions, and tolerability of nemonoxacin are similar to those of fluoroquinolones, recommending it as an alternative treatment for CAP (29). Additionally, pharmacokinetic and pharmacodynamic studies have demonstrated that nemonoxacin capsules (500 mg, po, q24h) exhibit significant efficacy in treating CAP caused by *S. pneumoniae*, *S. aureus*, and *M. pneumoniae*, with efficacy unaffected by food, gender, or mild renal impairment. For CAP caused by *Haemophilus parainfluenzae*, the 750 mg, q24h treatment regimen showed superior efficacy compared to the 500 mg dosage (30).

In the treatment of outpatients with mild to moderate CAP, the use of nemonoxacin (500 mg, po, qd, for a treatment duration of 8.24 ± 3.73 days) effectively improves clinical symptoms, with the advantages of good tolerability, few adverse reactions, low medical costs, and short treatment duration (31). Additionally, compared with levofloxacin and moxifloxacin, nemonoxacin also demonstrates economic advantages in the treatment of CAP, further supporting its role as a safe and effective treatment option for CAP (32).

### 4.2 The diagnostic value in differentiating tuberculosis

Tuberculosis is a chronic infectious disease caused by *M. tuberculosis*, which can affect many organs, with pulmonary tuberculosis being the most common (33–35). Its clinical manifestations are quite similar to those of CAP, making it difficult to distinguish between the two diseases, thereby leading to challenges in differential diagnosis (36–38). Studies have shown that the misdiagnosis rate of pulmonary tuberculosis among CAP patients in China is 6%, significantly higher than the Asian average of 3.30% (32). Traditional fluoroquinolones (such as levofloxacin and moxifloxacin), due to their antibacterial activity against *M. tuberculosis*, may mask the etiological characteristics of pulmonary tuberculosis during the treatment of CAP, thus delaying diagnosis and increasing medical costs (39, 40). In contrast, studies conducted by Zhao et al. (32) have demonstrated that nemonoxacin exhibits extremely weak inhibitory effects on *M. tuberculosis*. This characteristic endows it with potential advantages in distinguishing pulmonary tuberculosis from CAP—it may reduce the masking of the etiological features of pulmonary tuberculosis, thereby improving the accuracy of differential diagnosis, saving treatment costs, and even showing promise as a preferred empirical treatment option for patients with suspected pulmonary tuberculosis complicated by pulmonary infections (32). However, nemonoxacin also has obvious limitations: studies have shown that it has a high MIC value against *M. tuberculosis* (including multidrug-resistant strains) and its antibacterial activity is weaker than that of levofloxacin and moxifloxacin, which restricts its use in the treatment of tuberculosis patients (41). Therefore, although nemonoxacin’s unique antibacterial spectrum—including its activity against atypical pathogens and weak baseline activity against *M. tuberculosis*—theoretically provides room for exploring its application in “differential diagnosis” or “targeted therapy,” the current evidence is still insufficient to support its clinical use in such scenarios. In the future, there is an urgent need to conduct well-designed prospective studies (for example, in areas with a high burden of tuberculosis, enrolling cohorts of patients with undiagnosed respiratory symptoms and prospectively evaluating indicators such as the rate of delayed tuberculosis diagnosis and diagnostic accuracy after treatment with nemonoxacin). These studies will clarify its actual value and risks in this complex context, and provide reliable scientific evidence for whether and how to integrate it into diagnostic or treatment pathways.

### 4.3 Applications in other infectious diseases

Since nemonoxacin is widely distributed in various tissues and body fluids, it not only has excellent application value in the treatment of adult CAP and the differential diagnosis of tuberculosis, but also demonstrates significant clinical advantages in treating infectious diseases such as diabetic foot infection (DFI), urinary tract infection (UTI), bone infections, and acute exacerbation of chronic obstructive pulmonary disease (AECOPD) (Table 2).

**Table 2:** Application of nemonoxacin in infectious diseases.

Indications	Recommended dosage and course of treatment	Pharmacokinetic characteristics	Clinical efficacy and advantages	References
CAP	500 mg, po/ivgtt, qd 7–14 d	The concentration of alveolar epithelial liner fluid can be more than four times that of plasma	The clinical efficacy, safety, success rate of microbial treatment and tolerance are all good It is recommended that it can be used as an alternative treatment drug for CAP The therapeutic effect is not affected by mild renal function impairment	(12, 28, 29)
DFI	750 mg, po, qd 7–14 d	The drug concentration at the infection site (skin and soft tissue) is more than 2.5 times that of plasma The permeability of deep tissues is excellent	The clinical and microbiological efficacy is good Excellent tolerance	(44)
AECOPD	500 mg, po, qd 6–9 d	The concentration of alveolar epithelial liner fluid can be more than four times that of plasma	Shorten the remission time of clinical symptoms Prolong the interval period between the deterioration of moderate and severe conditions It can be used as an initial anti-infection treatment drug option for outpatients without the risk of *P. aeruginosa* infection	(51, 52)
UTI	Simple UTI: 500 mg, po, qd, 3 d Complexity UTI: 500 mg, po, qd, 14 d	The peak concentration of the drug in urine is more than 30 times that in blood Within 72 h, 60%–70% of the original form is excreted through the kidneys	The clinical efficacy, safety and tolerability are good It has strong antibacterial activity against *E. coli*. It can be used as a second-line option for treatment failure with other quinolone drugs	(56)
Patients with viral pneumonia accompanied by breathing difficulties and hypoxia	750 mg, qd	/	It is used when the treatment with fluoroquinolone drugs (levofloxacin/moxifloxacin) + arbitol does not improve	(65)
Bacterial keratitis	/	The protein binding rate is low, approximately 16%	The low protein binding rate may make it an option for local treatment	(66)

DFI is one of the most severe complications in diabetic patients, typically caused by various pathogens, especially staphylococci, and anti-infective therapy is the cornerstone of its treatment (42, 43). An open-label, single-arm, multicenter clinical trial has demonstrated that nemonoxacin (750 mg, po, qd, for 7–14 days) achieves excellent clinical and microbiological efficacy in treating DFI patients, with outstanding tolerability; pharmacokinetic studies further revealed that nemonoxacin exhibits significantly better permeability at the infection site (skin and soft tissue) compared to plasma distribution, with local drug concentrations reaching more than 2.5 times the plasma concentration, providing a pharmacological advantage for treating deep tissue infections (44).

Chronic obstructive pulmonary disease (COPD) is a heterogeneous lung condition typically characterized by persistent and progressively worsening airflow obstruction (45–47). AECOPD can lead to high morbidity and mortality, and anti-infective therapy is one of the crucial treatment modalities for patients with AECOPD showing signs of bacterial infection (48–50). For outpatient AECOPD patients without the risk of *Pseudomonas aeruginosa* infection, oral nemonoxacin has been recommended as one of the significant options for initial anti-infective therapy (51). The clinical study conducted by Meng et al. (52) further confirmed that in outpatient AECOPD patients, oral administration of nemonoxacin (500 mg, qd, for 6–9 days) significantly shortened the time to clinical symptom relief and effectively prolonged the interval to the next moderate to severe exacerbation compared to moxifloxacin (400 mg, qd, for 6–9 days).

UTI is one of the most common infectious diseases, usually caused by pathogenic G^−^ bacilli, especially *Escherichia coli* (53–55). Nemonoxacin exhibits good antibacterial activity against this pathogen and has high oral bioavailability. Following a single oral dose, approximately 60%–70% is excreted unchanged by the kidneys within 72 h, with peak drug concentrations in urine reaching 150–299 mg/L, which is more than 30 times higher than that in the blood. This makes nemonoxacin a potential preferred drug for the treatment of UTI (56). The prospective, open-label, multicenter study conducted by Du et al. (56) further confirmed that nemonoxacin (500 mg, po, qd, for 3 or 14 consecutive days) achieved favorable clinical efficacy, safety, and tolerability in the treatment of adult outpatients with acute lower UTI. Based on its pharmacodynamic properties and clinical evidence, a 3-day short-course therapy is recommended for uncomplicated UTI and recurrent UTI, while a prolonged 14-day course is advised for complicated UTI. Additionally, nemonoxacin can serve as a second-line treatment option when other quinolones exhibit suboptimal efficacy or treatment failure, providing a new strategy for the antimicrobial therapy of urinary tract infections.

In addition, studies have confirmed that nemonoxacin can also serve as an alternative treatment for infectious diseases such as viral pneumonia accompanied by dyspnea and hypoxia, and local treatment of bacterial keratitis (Table 2).

## 5 Application in patients with hepatic or renal insufficiency

Although 60%–70% of nemonoxacin is primarily excreted through the kidneys, it is not metabolized by P450 enzymes, and its metabolites in the liver are extremely low. However, with the in-depth research and widespread clinical application of nemonoxacin in various infectious diseases, special attention should be paid to the adjustment of its dosing regimen in patients with hepatic or renal insufficiency. The study by Yuan et al. (12) demonstrated that nemonoxacin (500 mg, po, qd, for 7–10 days) is safe and reliable in the treatment of CAP in elderly patients (60–70 years old), patients with renal impairment (creatinine clearance > 50 mL/min), and patients with mild to moderate hepatic impairment, without the need for dose adjustment. The “Guidelines for the Clinical Application of Quinolone Antibacterials in Emergency Medicine” recommend that no dose adjustment is necessary for patients with mild renal insufficiency when using nemonoxacin; it also states that for drugs primarily excreted by the kidneys, no dose adjustment is required in patients with impaired liver function (57). A single-dose, open-label, non-randomized, parallel clinical trial study demonstrated that extending the dosing interval of nemonoxacin (500 mg, po, q48h) achieves optimal efficacy in cases of severe renal impairment (58). Studies on patients with mild to moderate hepatic impairment showed that the use of nemonoxacin (500 mg, po, qd) had similar clinical efficacy and safety, as well as comparable systemic exposure, when compared to healthy subjects (59). Therefore, it is recommended that no dose adjustment is required for anti-infective treatment in such patients. For patients with severe hepatic insufficiency, there are currently no definitive clinical study results available, and further clinical validation is still needed.

## 6 Limitations of nemonoxacin in clinical application

Nemonoxacin demonstrates relatively favorable safety and tolerability profiles, though it may still elicit adverse drug reactions. The most frequently observed adverse effects manifest in the gastrointestinal and nervous systems. Furthermore, while structural modifications have enhanced its antibacterial spectrum and reduced the risk of drug-resistant mutations to some extent, clinical applications of nemonoxacin remain subject to certain limitations. Studies have shown that although nemonoxacin exhibits strong activity against PRSP and MRSA, it has obvious limitations. Firstly, its activity against ciprofloxacin-resistant strains and hospital-acquired MRSA isolates is significantly reduced (60), and its MIC90 against MRSA is significantly higher than that of tigecycline, eravacycline, tedizolid, and trimethoprim-sulfamethoxazole (4). Secondly, its antibacterial activity against *E. coli*, *Klebsiella pneumoniae*, *Acinetobacter baumannii*, and *P. aeruginosa* is weaker than that of levofloxacin (60). Similarly to ciprofloxacin and cefpirome, it also shows higher MIC values and lower sensitivity to ertapenem-insensitive strains (61). Thirdly, although nemonoxacin requires more mutation steps to induce resistance in *S. pneumoniae*, its antibacterial activity still decreases by 8-fold after three induction steps (9). Moreover, the resistance mutations only occur in the GyrA/GyrB/ParE genes and do not involve the ParC gene targeted by other fluoroquinolones (such as ciprofloxacin) (9), which means it cannot completely avoid the risk of resistance.

Furthermore, it is worth noting that although nemonoxacin shows certain advantages in antibacterial activity against G^+^ bacteria such as MRSA, PRSP, and VRE (14, 15), as well as atypical pathogens (62), *C. difficile* (18), and *M. abscessus* (20, 63), the manifestation of these advantages may be limited by multiple factors. On one hand, its relevant *in vitro* studies (16, 25), Phase II/III clinical trials (28), and post-marketing evaluations (12) are mainly concentrated in China (e.g., Taiwan) and South Africa. The limitation in the scope of research may lead to insufficient universality of its antibacterial activity advantages, making it difficult to directly promote to other regions globally. On the other hand, due to significant differences in bacterial resistance backgrounds across different regions, the activity advantages it demonstrates in specific regions may not be reproduced in other resistant environments. Therefore, in the future, it will be necessary to expand the verification scope through multi-center studies, deepen research on resistance mechanisms to cope with different resistance backgrounds, and expand application data in special populations, so as to further consolidate its clinical status and promote its rational application on a global scale.

## 7 Conclusion and outlook

In summary, the successful development of nemonoxacin as a novel non-fluorinated quinolone antibacterial agent marks a breakthrough in the clinical application of quinolone drugs. Through structural optimization strategies, it significantly enhances the inhibitory effect on G^+^ bacteria while retaining the broad-spectrum antibacterial activity of traditional fluoroquinolones. It also reduces the occurrence of fluorine-related adverse reactions, lowers the mutation rate of bacterial resistance, and exerts immunomodulatory effects on the body, thereby demonstrating high clinical application value in various infectious diseases. Clinical studies have fully validated the efficacy, safety, and potential clinical value of nemonoxacin in diseases such as CAP, DFI, AECOPD, and UTI, particularly in the differential diagnosis of tuberculosis. Moreover, for patients with hepatic or renal insufficiency, existing evidence supports that no dose adjustment is required for those with mild to moderate impairment, while patients with severe renal impairment can achieve individualized treatment by extending the dosing interval, further expanding its clinical applicability.

In the future, we believe that the research direction of nemonoxacin can focus on the following aspects: First, further deepen the study of its mechanisms of action, such as bacterial resistance mechanisms and immune regulation mechanisms, clarify its pathways of action, propose response strategies, and delay the occurrence of bacterial resistance; second, continue to conduct clinical trials and large-scale real-world studies, deeply explore its clinical applications in infectious diseases such as lung and bronchial infections, skin and soft tissue infections, bone and joint infections, and abdominal infections, and evaluate the drug’s clinical efficacy, safety, and tolerability in special populations (children, pregnant women, patients with severe renal impairment, immunocompromised patients), complex infections (such as multidrug-resistant bacterial infections, biofilm-associated infections), and high doses (750 mg), and optimize the dosing regimen by combining pharmacokinetic/pharmacodynamic (PK/PD) models, providing more data support for expanding clinical indications and promoting precision medicine practices, thereby enhancing the clinical application value of the drug; third, improve drug formulations and administration methods, such as inhalation, topical eye drops, and local injections, to directly act on the infection site, increase local drug concentration, enhance efficacy, and reduce adverse reactions from systemic administration; finally, conduct pharmacoeconomic studies to evaluate its cost-effectiveness ratio and cost–benefit ratio in different treatment scenarios, providing a scientific basis for the formulation of medical insurance payment policies, clinical drug selection, and public health policy making. With ongoing research, nemonoxacin is expected to become a crucial option in the field of anti-infective therapy, offering a new solution to address the global crisis of drug-resistant bacteria.

## References

[1] Chung DT Tsai CY Chen SJ Chang LW King CH Hsu CH. Multiple-dose safety, tolerability, and pharmacokinetics of oral nemonoxacin (TG-873870) in healthy volunteers. Antimicrob Agents Chemother. (2010) 54:411–7. doi: 10.1128/AAC.00683-09, PMID: 19884374 PMC2798523

[2] Poole RM. Nemonoxacin: first global approval. Drugs. (2014) 74:1445–53. doi: 10.1007/s40265-014-0270-0, PMID: 25079302

[3] Rusu A Lungu IA Moldovan OL Tanase C Hancu G. Structural characterization of the millennial antibacterial (Fluoro)quinolones-shaping the fifth generation. Pharmaceutics. (2021) 13:13. doi: 10.3390/pharmaceutics13081289, PMID: 34452252 PMC8399897

[4] Ding L Yang Y Zheng C Sun G Han R Guo Y. Activities of eravacycline, tedizolid, norvancomycin, nemonoxacin, ceftaroline, and comparators against 1, 871 Staphylococcus and 1, 068 *Enterococcus* species isolates from China: updated report of the CHINET study 2019. Microbiol Spectr. (2022) 10:e0171522. doi: 10.1128/spectrum.01715-22, PMID: 36326536 PMC9769667

[5] Kocsis B Domokos J Szabo D. Chemical structure and pharmacokinetics of novel quinolone agents represented by avarofloxacin, delafloxacin, finafloxacin, zabofloxacin and nemonoxacin. Ann Clin Microbiol Antimicrob. (2016) 15:34. doi: 10.1186/s12941-016-0150-4, PMID: 27215369 PMC4878067

[6] Cheng SL Wu RG Chuang YC Perng WC Tsao SM Chang YT. Integrated safety summary of phase II and III studies comparing oral nemonoxacin and levofloxacin in community-acquired pneumonia. J Microbiol Immunol Infect. (2019) 52:743–51. doi: 10.1016/j.jmii.2018.11.006, PMID: 30616912

[7] Li X Chen Y Xu X Li Y Fan Y Liu X. Pharmacokinetics and pharmacodynamics of nemonoxacin in a neutropenic murine lung infection model against *Streptococcus pneumoniae*. Front Pharmacol. (2021) 12:658558. doi: 10.3389/fphar.2021.658558, PMID: 34017256 PMC8129567

[8] Qin X Huang H. Review of nemonoxacin with special focus on clinical development. Drug Des Devel Ther. (2014) 8:765–74. doi: 10.2147/DDDT.S63581, PMID: 25045247 PMC4094567

[9] Roychoudhury S Makin K Twinem T Leunk R Hsu MC. In vitro resistance development to nemonoxacin in *Streptococcus pneumoniae*: a unique profile for a novel nonfluorinated quinolone. Microb Drug Resist. (2016) 22:578–84. doi: 10.1089/mdr.2016.0021, PMID: 27267788 PMC5073217

[10] Chen N Li X Guo B Zou J Lin D Li X. Nemonoxacin has immunoprotective effects on reducing mortality in lipopolysaccharide-induced mouse Sepsis model. Inflammation. (2020) 43:2276–86. doi: 10.1007/s10753-020-01296-9, PMID: 32661821

[11] Huang CH Lai CC Chen YH Hsueh PR. The potential role of nemonoxacin for treatment of common infections. Expert Opin Pharmacother. (2015) 16:263–70. doi: 10.1517/14656566.2015.978288, PMID: 25529577

[12] Yuan J Zhang X Chen J Zhang Y Zhu F Huang H. Safety of oral nemonoxacin: a systematic review of clinical trials and postmarketing surveillance. Front Pharmacol. (2022) 13:1067686. doi: 10.3389/fphar.2022.1067686, PMID: 36569296 PMC9780658

[13] Yang JJ Cheng A Tai HM Chang LW Hsu MC Sheng WH. Selected mutations by nemonoxacin and fluoroquinolone exposure among relevant gram-positive bacterial strains in Taiwan. Microb Drug Resist. (2020) 26:110–7. doi: 10.1089/mdr.2019.0048, PMID: 31478786

[14] Li CR Li Y Li GQ Yang XY Zhang WX Lou RH. In vivo antibacterial activity of nemonoxacin, a novel non-fluorinated quinolone. J Antimicrob Chemother. (2010) 65:2411–5. doi: 10.1093/jac/dkq341, PMID: 20858687

[15] Huang J Guo S Li X Yuan F Li Y Xu B. Nemonoxacin enhances antibacterial activity and anti-resistance mutation ability of vancomycin against methicillin-resistant *Staphylococcus aureus* in an in vitro dynamic pharmacokinetic/Pharmacodynamic model. Antimicrob Agents Chemother. (2022) 66:e0180021. doi: 10.1128/AAC.01800-21, PMID: 34902266 PMC8846321

[16] Chen YH Liu CY Lu JJ King CH Hsueh PR. In vitro activity of nemonoxacin (TG-873870), a novel non-fluorinated quinolone, against clinical isolates of *Staphylococcus aureus*, enterococci and *Streptococcus pneumoniae* with various resistance phenotypes in Taiwan. J Antimicrob Chemother. (2009) 64:1226–9. doi: 10.1093/jac/dkp370, PMID: 19833635

[17] Wang N Chen Y Qu X Bian X Hu J Xu X. In vitro pharmacodynamics of nemonoxacin and other antimicrobial agents against *Mycoplasma pneumoniae*. Microbiol Spectr. (2023) 11:e0243123. doi: 10.1128/spectrum.02431-23, PMID: 37975686 PMC10715200

[18] Lee CC Yan XZ Wu HT Ko WC Tsai PJ Hung YP. Potential effectiveness of parenteral nemonoxacin in the treatment of clostridioides difficile infections: in vitro, ex vivo, and mouse studies. Front Microbiol. (2024) 15:1418817. doi: 10.3389/fmicb.2024.1418817, PMID: 39228379 PMC11368742

[19] Liao CH Ko WC Lu JJ Hsueh PR. Characterizations of clinical isolates of *Clostridium difficile* by toxin genotypes and by susceptibility to 12 antimicrobial agents, including fidaxomicin (OPT-80) and rifaximin: a multicenter study in Taiwan. Antimicrob Agents Chemother. (2012) 56:3943–9. doi: 10.1128/AAC.00191-12, PMID: 22508299 PMC3393409

[20] Jiang GL Wang F Xue Y Jia JN Huang HR. In vitro evaluation of the antibacterial activity of nemonoxacin against *Mycobacterium tuberculosis*, mycobacterium intracellulare and *Mycobacterium abscessus*. Zhonghua Jie He He Hu Xi Za Zhi. (2020) 43:1061–5. doi: 10.3760/cma.j.cn112147-20200813-00896, PMID: 33333640

[21] Yang JC Lee PI Hsueh PR. In vitro activity of nemonoxacin, tigecycline, and other antimicrobial agents against *Helicobacter pylori* isolates in Taiwan, 1998-2007. Eur J Clin Microbiol Infect Dis. (2010) 29:1369–75. doi: 10.1007/s10096-010-1009-9, PMID: 20658256

[22] Lai CC Liu WL Ko WC Chen YH Tan HR Huang YT. Multicenter study in Taiwan of the in vitro activities of nemonoxacin, tigecycline, doripenem, and other antimicrobial agents against clinical isolates of various Nocardia species. Antimicrob Agents Chemother. (2011) 55:2084–91. doi: 10.1128/AAC.01808-10, PMID: 21343461 PMC3088233

[23] Lai CC Tan CK Lin SH Liao CH Chou CH Hsu HL. Comparative in vitro activities of nemonoxacin, doripenem, tigecycline and 16 other antimicrobials against *Nocardia brasiliensis*, Nocardia asteroides and unusual Nocardia species. J Antimicrob Chemother. (2009) 64:73–8. doi: 10.1093/jac/dkp144, PMID: 19398458

[24] Chen YH Liu CY Ko WC Liao CH Lu PL Huang CH. Trends in the susceptibility of methicillin-resistant *Staphylococcus aureus* to nine antimicrobial agents, including ceftobiprole, nemonoxacin, and tyrothricin: results from the Tigecycline in vitro surveillance in Taiwan (TIST) study, 2006-2010. Eur J Clin Microbiol Infect Dis. (2014) 33:233–9. doi: 10.1007/s10096-013-1949-y, PMID: 23955154

[25] Chen P Ho MW Lu PL Tang HJ Sy CL Wang JT. Taiwan *Staphylococcus aureus*: comparative in vitro antibacterial activity of nemonoxacin and other fluoroquinolones in correlation with resistant mechanisms in contemporary methicillin-resistant *Staphylococcus aureus* blood isolates in Taiwan. Ann Clin Microbiol Antimicrob. (2025) 24:5. doi: 10.1186/s12941-024-00772-6, PMID: 39825371 PMC11742215

[26] Zhang YX Chen NY Feng MQ Lin DF Huang JW Zhao X. Investigation into the specific immunomodulatory effects of nemonoxacin onmacrophages. Chin J Infect Chemother. (2020):20. doi: 10.16718/j.1009-7708.2020.04.001

[27] Aliberti S Dela Cruz CS Amati F Sotgiu G. Restrepo MI. Restrepo: community-acquired pneumonia. Lancet. (2021) 398:906–19. doi: 10.1016/S0140-6736(21)00630-9, PMID: 34481570

[28] Li Y Zhu D Sun S Chang X Cao Z Yang Y. A multicentre, randomised, double-blind, double-dummy, parallel-controlled, phase 3 clinical trial assessing the efficacy and safety of intravenous nemonoxacin malate vs. levofloxacin for community-acquired pneumonia in adult patients. Int J Antimicrob Agents. (2024) 64:107235. doi: 10.1016/j.ijantimicag.2024.107235, PMID: 38851462

[29] Khan AS Iqbal A Muhammad AA Mazhar F Lodhi MF Ahmed KF. Safety and efficacy of nemonoxacin vs levofloxacin in patients with community-acquired pneumonia: a systematic review and meta-analysis of randomized control trials. Cureus. (2023) 15:e37650. doi: 10.7759/cureus.37650, PMID: 37200652 PMC10188129

[30] Chen Y Wu X Tsai C Chang L Yu J Cao G. Integrative population pharmacokinetic/pharmacodynamic analysis of nemonoxacin capsule in Chinese patients with community-acquired pneumonia. Front Pharmacol. (2023) 14:912962. doi: 10.3389/fphar.2023.912962, PMID: 36923351 PMC10010492

[31] Zhao B Yu X Chen R Zheng R. Efficacy and safety of nemonoxacin in outpatients with community-acquired pneumonia. Infect Drug Resist. (2020) 13:2099–104. doi: 10.2147/IDR.S248092, PMID: 32669862 PMC7337426

[32] Zhao M Chi Z Pan X Yin Y Tang W. Economic evaluation of nemonoxacin, moxifloxacin and levofloxacin in the treatment of early community-acquired pneumonia with possible pulmonary tuberculosis. Int J Environ Res Public Health. (2022) 19:19. doi: 10.3390/ijerph19084816, PMID: 35457683 PMC9028707

[33] Grossman RF Hsueh PR Gillespie SH Blasi F. Community-acquired pneumonia and tuberculosis: differential diagnosis and the use of fluoroquinolones. Int J Infect Dis. (2014) 18:14–21. doi: 10.1016/j.ijid.2013.09.013, PMID: 24211230

[34] Acharya B Acharya A Gautam S Ghimire SP Mishra G Parajuli N. Advances in diagnosis of tuberculosis: an update into molecular diagnosis of *Mycobacterium tuberculosis*. Mol Biol Rep. (2020) 47:4065–75. doi: 10.1007/s11033-020-05413-7, PMID: 32248381

[35] Ehrt S Schnappinger D Rhee KY. Metabolic principles of persistence and pathogenicity in *Mycobacterium tuberculosis*. Nat Rev Microbiol. (2018) 16:496–507. doi: 10.1038/s41579-018-0013-4, PMID: 29691481 PMC6045436

[36] Dheda K Makambwa E Esmail A. The great masquerader: tuberculosis presenting as community-acquired pneumonia. Semin Respir Crit Care Med. (2020) 41:592–604. doi: 10.1055/s-0040-1710583, PMID: 32564347

[37] Kunimoto D Long R. Tuberculosis: still overlooked as a cause of community-acquired pneumonia--how not to miss it. Respir Care Clin N Am. (2005) 11:25–34. doi: 10.1016/j.rcc.2004.10.007, PMID: 15763219

[38] Shen GH Tsao TC Kao SJ Lee JJ Chen YH Hsieh WC. Does empirical treatment of community-acquired pneumonia with fluoroquinolones delay tuberculosis treatment and result in fluoroquinolone resistance in *Mycobacterium tuberculosis*? Controversies and solutions. Int J Antimicrob Agents. (2012) 39:201–5. doi: 10.1016/j.ijantimicag.2011.11.014, PMID: 22285045 PMC7127649

[39] Evans D Hirasen K Ramushu C Long L Sinanovic E Conradie F. Patient and provider costs of the new BPaL regimen for drug-resistant tuberculosis treatment in South Africa: a cost-effectiveness analysis. PLoS One. (2024) 19:e0309034. doi: 10.1371/journal.pone.0309034, PMID: 39432463 PMC11493257

[40] Mafirakureva N Mukherjee S de Souza M Kelly-Cirino C Songane MJP Cohn J. Cost-effectiveness analysis of interventions to improve diagnosis and preventive therapy for paediatric tuberculosis in 9 sub-Saharan African countries: a modelling study. PLoS Med. (2023) 20:e1004285. doi: 10.1371/journal.pmed.1004285, PMID: 37672524 PMC10511115

[41] Tan CK Lai CC Liao CH Chou CH Hsu HL Huang YT. Comparative in vitro activities of the new quinolone nemonoxacin (TG-873870), gemifloxacin and other quinolones against clinical isolates of *Mycobacterium tuberculosis*. J Antimicrob Chemother. (2009) 64:428–9. doi: 10.1093/jac/dkp174, PMID: 19454523

[42] Liu W Song L Sun W Fang W Wang C. Distribution of microbes and antimicrobial susceptibility in patients with diabetic foot infections in South China. Front Endocrinol. (2023) 14:1113622. doi: 10.3389/fendo.2023.1113622, PMID: 36761201 PMC9904418

[43] Baig MS Banu A Zehravi M Rana R Burle SS Khan SL. An overview of diabetic foot ulcers and associated problems with special emphasis on treatments with antimicrobials. Life. (2022) 12:1054. doi: 10.3390/life12071054, PMID: 35888142 PMC9316721

[44] Lipsky BA Ganib M Rogers LC Hwang JS Tsaie CY Chang LW. A pilot study of nemonoxacin in patients with diabetic foot infections. J Infect Dis Therapy. (2019) 7:397. doi: 10.4172/2332-0877.1000397

[45] Vestbo J Hurd SS Agusti AG Jones PW Vogelmeier C Anzueto A. Global strategy for the diagnosis, management, and prevention of chronic obstructive pulmonary disease: GOLD executive summary. Am J Respir Crit Care Med. (2013) 187:347–65. doi: 10.1164/rccm.201204-0596PP, PMID: 22878278

[46] Christenson SA Smith BM Bafadhel M Putcha N. Chronic obstructive pulmonary disease. Lancet. (2022) 399:2227–42. doi: 10.1016/S0140-6736(22)00470-6, PMID: 35533707

[47] Labaki WW Rosenberg SR. Chronic obstructive pulmonary disease. Ann Intern Med. (2020) 173:ITC17-ITC32. doi: 10.7326/AITC202008040, PMID: 32745458

[48] Ritchie AI Wedzicha JA. Definition, causes, pathogenesis, and consequences of chronic obstructive pulmonary disease exacerbations. Clin Chest Med. (2020) 41:421–38. doi: 10.1016/j.ccm.2020.06.007, PMID: 32800196 PMC7423341

[49] Qian Y Cai C Sun M Lv D Zhao Y. Analyses of factors associated with acute exacerbations of chronic obstructive pulmonary disease: a review. Int J Chron Obstruct Pulmon Dis. (2023) 18:2707–23. doi: 10.2147/COPD.S433183, PMID: 38034468 PMC10683659

[50] Medrinal C Bonnevie T. Physiotherapy during and after acute exacerbation of COPD. Rev Mal Respir. (2022) 39:386–97. doi: 10.1016/j.rmr.2022.02.056, PMID: 35221161

[51] Chronic obstructive pulmonary disease Group of Chinese Thoracic Society; chronic obstructive pulmonary disease Committee of Chinese Association of chest physician. Guidelines for the diagnosis and management of chronic obstructive pulmonary disease (revised version 2021). Zhonghua Jie He He Hu Xi Za Zhi. (2021) 44:170–205. doi: 10.3760/cma.j.cn112147-20210109-00031, PMID: 33721932

[52] Meng W Zeng H Zhao Z Xiong R Chen Y Li Z. Nemonoxacin achieved a better symptomatic improvement and a prolonged interval to next exacerbation than moxifloxacin for outpatients with acute exacerbations of chronic obstructive pulmonary disease. Sci Rep. (2023) 13:16954. doi: 10.1038/s41598-023-44188-2, PMID: 37805617 PMC10560244

[53] Byron JK. Urinary tract infection. Vet Clin North Am Small Anim Pract. (2019) 49:211–21. doi: 10.1016/j.cvsm.2018.11.005, PMID: 30591189

[54] Schmiemann G Kranz J Mandraka F Schubert S Wagenlehner F Gagyor I. The diagnosis, treatment, and prevention of recurrent urinary tract infection. Dtsch Arztebl Int. (2024) 121:373–82. doi: 10.3238/arztebl.m2024.0068, PMID: 38686602 PMC11539874

[55] Chenoweth CE. Urinary tract infections: 2021 update. Infect Dis Clin N Am. (2021) 35:857–70. doi: 10.1016/j.idc.2021.08.003, PMID: 34752223

[56] Du Z Zheng B Chen S Cui L Wu H Gao Z. Clinical utility of oral nemonoxacin 500 mg once daily for the treatment of acute lower urinary tract infections: a prospective open-label, multicenter study. BMC Infect Dis. (2025) 25:501. doi: 10.1186/s12879-025-10915-5, PMID: 40211176 PMC11987325

[57] Emergency Physicians Branch of Chinese Medical Doctor Association, Emergency Medicine Branch of Chinese Medical Association, China Emergency Specialized Medical Association, Beijing Emergency Medicine Society, Zhao XD, Lv CZ, et al. Guidelines for the emergency clinical application of quinolone antibacterial drugs. Chin Emerg Med. (2020) 40:1047–56. doi: 10.3969/j.issn.1002-1949.2020.11.004

[58] Li Y Lu J Kang Y Xu X Li X Chen Y. Nemonoxacin dosage adjustment in patients with severe renal impairment based on population pharmacokinetic and pharmacodynamic analysis. Br J Clin Pharmacol. (2021) 87:4636–47. doi: 10.1111/bcp.14881, PMID: 33928669

[59] Kang Y Li Y Xu F Zhang J Wang K Chen Y. Population pharmacokinetics study of nemonoxacin among Chinese patients with moderate hepatic impairment. Clin Ther. (2019) 41:505–517.e0. doi: 10.1016/j.clinthera.2019.01.015, PMID: 30819510

[60] Lai CC Lee KY Lin SW Chen YH Kuo HY Hung CC. Nemonoxacin (TG-873870) for treatment of community-acquired pneumonia. Expert Rev Anti-Infect Ther. (2014) 12:401–17. doi: 10.1586/14787210.2014.894881, PMID: 24579813

[61] Hsu MS Liao CH Liu CY Yang CJ Huang YT Hsueh PR. In vitro susceptibilities of clinical isolates of ertapenem-non-susceptible Enterobacteriaceae to nemonoxacin, tigecycline, fosfomycin and other antimicrobial agents. Int J Antimicrob Agents. (2011) 37:276–8. doi: 10.1016/j.ijantimicag.2010.12.003, PMID: 21269811

[62] Wang N Liu W Zhou Y Liu Y. In vitro activities of nemonoxacin and other antimicrobial agents against human *Mycoplasma* and *Ureaplasmas* isolates and their defined resistance mechanisms. Front Microbiol. (2019) 10:1890. doi: 10.3389/fmicb.2019.01890, PMID: 31456791 PMC6700270

[63] Li B Guo Q Chu HQ. In vitro evaluation of the antibacterial activity of nemonoxacin against *Mycobacterium abscessus*. Zhonghua Jie He He Hu Xi Za Zhi. (2021) 44:947–52. doi: 10.3760/cma.j.cn112147-20210507-00311, PMID: 34758520

[64] Zhao C Lv Y Li X Hou F Ma X Wei M. Effects of nemonoxacin on thorough ECG QT/QTc interval: a randomized, placebo-and positive-controlled crossover study in healthy Chinese adults. Clin Ther. (2018) 40:983–92. doi: 10.1016/j.clinthera.2018.04.014, PMID: 29803534

[65] Zhang J Zhou L Yang Y Peng W Wang W Chen X. Therapeutic and triage strategies for 2019 novel coronavirus disease in fever clinics. Lancet Respir Med. (2020) 8:e11–2. doi: 10.1016/S2213-2600(20)30071-0, PMID: 32061335 PMC7159020

[66] Herbert R Caddick M Somerville T McLean K Herwitker S Neal T. Potential new fluoroquinolone treatments for suspected bacterial keratitis. BMJ Open Ophthalmol. (2022) 7:e001002. doi: 10.1136/bmjophth-2022-001002, PMID: 36161851 PMC9297210

